# Pigment epithelium-derived factor promotes tumor metastasis through an interaction with laminin receptor in hepatocellular carcinomas

**DOI:** 10.1038/cddis.2017.359

**Published:** 2017-08-03

**Authors:** Jianjing Hou, Chao Ge, Meiling Cui, Tengfei Liu, Xiaoqin Liu, Hua Tian, Fangyu Zhao, Taoyang Chen, Ying Cui, Ming Yao, Jinjun Li, Hong Li

**Affiliations:** 1 State Key Laboratory of Oncogenes and Related Genes, Shanghai Cancer Institute, Renji Hospital, Shanghai Jiaotong University School of Medicine, Shanghai 200032, China; 2 Heze Municipal Hospital, Shandong 274031, China; 3 Department of Etiology, Qi Dong Liver Cancer Institute, Qi Dong 226200, Jiangsu Province, China; 4 Cancer Institute of Guangxi, Nanning 530027, China

## Abstract

Pigment epithelium-derived factor (PEDF) has complex functions in tumor metastasis, but little is known about the roles of PEDF and its receptors in hepatocellular carcinoma (HCC). Here we found that high expression of PEDF is associated with shorter overall survival in HCC patients. Forced expression of PEDF enhanced HCC cell aggressive behavior *in vitro* and *in vivo*, whereas silencing PEDF expression reduced migration and invasion. Furthermore, PEDF expression led to changes in cell morphology and the expression of epithelial–mesenchymal transition (EMT)-related markers via ERK1/2 signaling pathway, including the upregulation of N-cadherin and slug, and the downregulation of E-cadherin in HCC cells. Our results further showed that PEDF could interact with laminin receptor (LR) and LR knockdown attenuated PEDF-induced migration, invasion and the change of EMT-related markers. More importantly, in clinical HCC specimens, we found that PEDF expression was correlated with subcellular localization of LR, and that high expression of PEDF and positive expression of LR predicted a poor prognosis. In conclusion, our results demonstrate a novel functional role of PEDF/LR axis in driving metastasis through ERK1/2-mediated EMT in HCC and provided a promising prognostic marker in HCC.

Hepatocellular carcinoma (HCC) is the third most common cause of cancer-associated death worldwide and remains a major public health challenge.^1, 2^ Accumulating evidence suggests that cancer metastasis is associated with a poor prognosis;^3^ therefore, uncovering the mechanisms underlying HCC metastasis may contribute to identifying therapeutic targets.

Pigment epithelium-derived factor (PEDF) is a member of the superfamily of serine protease inhibitors and was initially isolated from fetal human retinal pigment epithelial cells.^4, 5^ PEDF is highly expressed in the adipose tissue, liver, eye, heart, skeletal muscle, spleen, brain and bone.^6^ PEDF exerts a range of biological effects in many physiological and pathophysiological processes, including neuroprotection, fibrogenesis and inflammation.^7, 8^ Previous studies have shown that PEDF has complex and often opposing roles in cancer angiogenesis, tumor growth and metastasis. PEDF displays anti-tumor effects in different tumorous tissues, including pancreatic carcinomas,^9^ melanomas^10^ and ovarian cancer.^11^ Therefore, the potential use of PEDF in cancer therapeutics has generated much expectation.^12^ In contrast to the known function of PEDF in the tumor cells mentioned above, there is some controversy regarding the pathophysiological role of PEDF, as PEDF levels are increased in certain types of cancers,^13, 14^ and PEDF promotes the stemness and self-renewal of glioma stem cells.^15^ More importantly, the function of PEDF in HCC is a notable exception:^16^ PEDF levels are higher in HCC than in adjacent normal tissues and secreted PEDF are greater in serum samples from patients with HCC than those from normal controls, indicating that serum PEDF has a potential role as an auxiliary marker for HCC in diagnosis.^14, 17^ Moreover, increased expression of PEDF may exert anti-apoptotic effects in HCC by inhibiting the lysosomal degradation of Bcl-xL.^14^ Therefore, if PEDF is to be used for cancer therapeutics or as a diagnostic and prognostic marker, a deeper investigation of its functions and mechanisms in various cancers is required.

PEDF is a secretory glycoprotein and it interacts with its two putative receptors, namely adipose triglyceride lipase (ATGL) and the laminin receptor (LR).^12, 18, 19^ PEDF protects osteoblastic cells from glucocorticoid-induced apoptosis,^20^ increases vascular hyperpermeability^21^ and induces triglyceride degradation via ATGL,^22, 23^ and LR is reportedly involved in the anti-inflammatory and anti-thrombogenic effects of PEDF,^12, 19^ and anti-LRP/LR-specific antibody IgG1-iS18 impedes adhesion and invasion of liver cancer cells.^24^ However, how PEDF regulates HCC metastasis through its receptors is poorly understood.

In the present study, we found that PEDF was significantly associated with HCC progression and promoted HCC cell metastasis. In addition, we evaluated the relationship between PEDF and its receptors in HCC for the first time and showed that PEDF augmented cell invasion, migration and epithelial–mesenchymal transition (EMT) via binding to LR. Accordingly, PEDF expression was correlated with subcellular localization of LR, which predicts a poor prognosis in HCC. These findings demonstrate that PEDF has a positive role in regulating HCC progression.

## Results

### The high expression of PEDF is positively correlated with poor prognosis in HCC

**Figure 1:**
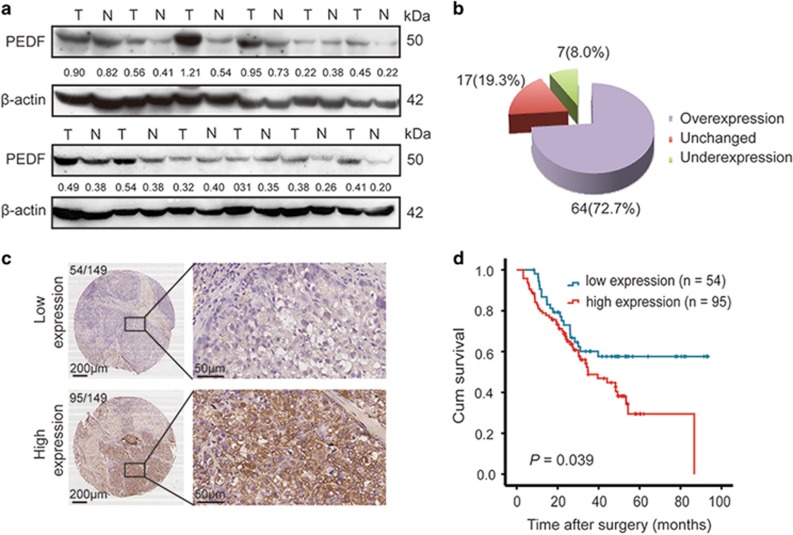
Clinical significance of PEDF in HCC. (**a**) Western blotting of PEDF expression level in HCC tissues and matched adjacent non-tumorous liver tissues. T, HCC tissues; N, adjacent non-tumorous liver tissues. (**b**) Statistical analysis of PEDF expression in HCC samples between HCC tissues and adjacent non-cancerous tissues. (**c**) IHC analysis of PEDF expression in 149 HCC samples. Representative images are shown. (**d**) OS analysis of HCC patients with PEDF expression (*n*=149)

Previous studies have shown that PEDF is commonly upregulated in HCC compared with adjacent normal tissues.^14^ We also assessed PEDF expression in HCC tissues and matched adjacent non-tumorous liver tissues from 88 patients by western blotting. Out of the 88 cases, 64 (72.7%) had higher PEDF protein expression in HCC tissues compared with the adjacent non-cancerous liver tissues, 17 (19.3%) had similar expression and only 7 (8.0%) had lower expression (Figures 1a and b). According to the results of immunohistochemical (IHC) staining for PEDF, 149 HCC patients were divided into 2 groups: a high-PEDF-expression group (score of 3 or 4) and a low-PEDF-expression group (weak or no staining, score of 0, 1 or 2) (Figure 1c). The Kaplan–Meier survival analysis revealed that higher levels of PEDF were associated with a shorter overall survival (OS) time (*P*=0.039; Figure 1d), suggesting that high level of PEDF expression may contribute to a poor HCC prognosis. However, there was no correlation between PEDF expression and clinicopathological factors, such as age, gender, tumor size, the presence of cirrhosis, serum *α*-fetoprotein and histological grade (Supplementary Table 1). The relationship between PEDF protein expression and the metastasis-related features (extra-hepatic metastasis, lymph node metastasis and venous invasion) were also analyzed (Supplementary Table 1). The results showed that two (100%) HCC patients with extra-hepatic metastasis and three (100%) cases with lymph node metastasis all had high expression level of PEDF. Out of the 16 cases with venous invasion, 12 cases (75%) had high PEDF protein expression in HCC tissues, but there were no significant differences and large-scale clinical validations were needed.

### PEDF increases the metastatic potential of HCC cells *in vitro* and *in vivo*

**Figure 2:**
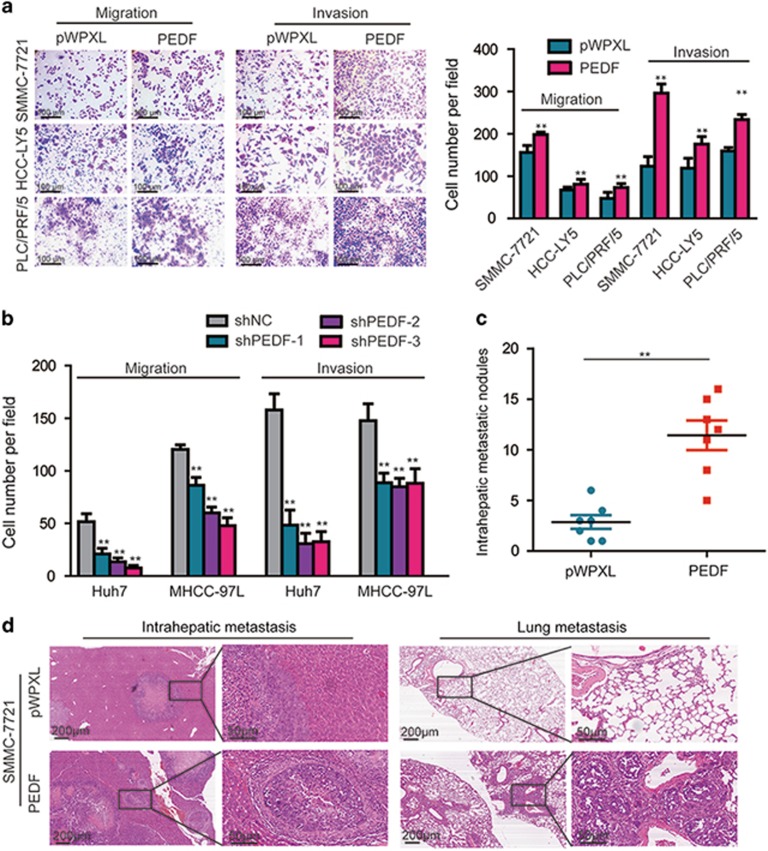
PEDF promotes HCC cell migration and invasion *in vitro* and *in vivo*. (**a**) The *in vitro* migration and invasion ability of SMMC-7721, HCC-LY5 and PLC/PRF/5 cells lentivirally transduced with PEDF or with vector (pWPXL) were assessed using a transwell assay. (**b**) The *in vitro* migration and invasion ability of Huh7 and MHCC-97L cells lentivirally transduced with shPEDF were assessed using a transwell assay; shNC was used as the control. (**c**) The numbers of intrahepatic metastatic nodules are shown in the panels. (**d**) Representative images of liver metastatic nodules and lung metastatic nodules derived from SMMC-7721 cells stably overexpressing PEDF or the control are presented. The statistical analyses compare the experimental group and the control group. *P<0.05 and **P<0.01

To verify the role of PEDF in HCC metastasis, we stably overexpressed PEDF in SMMC-7721, HCC-LY5 and PLC/PRF/5 cells, and knocked down the endogenous expression of PEDF in Huh7 and MHCC-97L cells using lentiviral infection (Supplementary Figure 1b–d) according to the endogenous expression levels of PEDF in HCC cells (Supplementary Figure 1a). Transwell assays demonstrated that PEDF overexpression significantly enhanced both the migration and invasion of SMMC-7721, HCC-LY5 and PLC/PRF/5 cells (Figure 2a), whereas silencing PEDF markedly inhibited both the migration and invasion of Huh7 and MHCC-97L cells (Figure 2b and Supplementary Figure 2a).

To investigate whether PEDF could affect HCC cell metastasis *in vivo*, SMMC-7721-PEDF and SMMC-7721-pWPXL cells were inoculated into the hepatic lobes of nude mice. After 4 weeks, PEDF-overexpressing cells formed larger tumors than control group (*P*<0.05; Supplementary Figure 3a), consistent with previous reports.^14^ Histological examinations of liver and lung tissues indicated that PEDF-overexpressing tumor-bearing mice had more intrahepatic metastatic nodules than control mice (*P*<0.01; Figures 2c and d). In addition, we found that three of seven mice in the experimental group developed lung metastases, whereas no mice developed lung metastases in the control group (Figure 2d). Therefore, these findings indicated that PEDF effectively facilitated HCC metastasis *in vitro* and *in vivo*.

### PEDF positively regulates EMT in HCC cells

**Figure 3:**
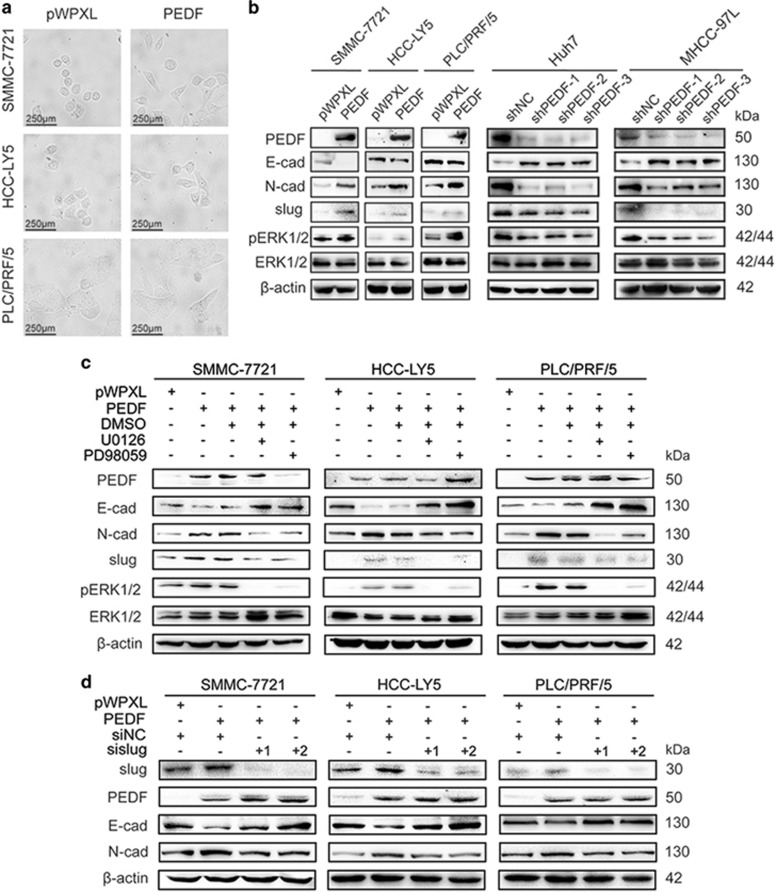
PEDF positively regulates EMT in HCC cells. (**a**) The images show morphologic changes in PEDF-overexpressing SMMC-7721, HCC-LY5 and PLC/PRF/5 cells and in the control. (**b**) The expression of E-cadherin, N-cadherin, slug, pERK1/2 and ERK1/2 was detected by western blotting in PEDF-overexpressing SMMC-7721, HCC-LY5 and PLC/PRF/5 cells, and PEDF knockdown Huh7 and MHCC-97L cells, and the corresponding controls. (**c**) Western blotting analysis showed that ERK1/2 inhibitor U0126 (10 *μ*M) and PD98059 (30 *μ*M) inhibited the activation of ERK1/2 signaling induced by PEDF, as well as the EMT-related markers in SMMC-7721, HCC-LY5 and PLC/PRF/5. (**d**) Western blotting analysis showed that slug knockdown inhibited the change of E-cadherin and N-cadherin induced by PEDF overexpression

Over the past decade, numerous studies have reported that EMT is required for metastasis in malignant cancer.^25, 26, 27^ During the process of EMT, epithelial cells lose their polarized organization and acquire invasive and migratory capabilities. Our results showed that PEDF overexpression led to cell morphology changes in SMMC-7721, HCC-LY5 and PLC/PRF/5 cells, suggesting the induction of EMT (Figure 3a). Consequently, the expression of EMT-related markers was measured using western blotting. PEDF overexpression caused a loss of the epithelial marker E-cadherin and increased the expression of the mesenchymal marker N-cadherin and the EMT-related transcription factor slug (Figure 3b). In contrast, the depletion expression of PEDF increased E-cadherin expression and decreased N-cadherin and slug expression in Huh7 and MHCC-97L cells (Figure 3b).

**Figure 4:**
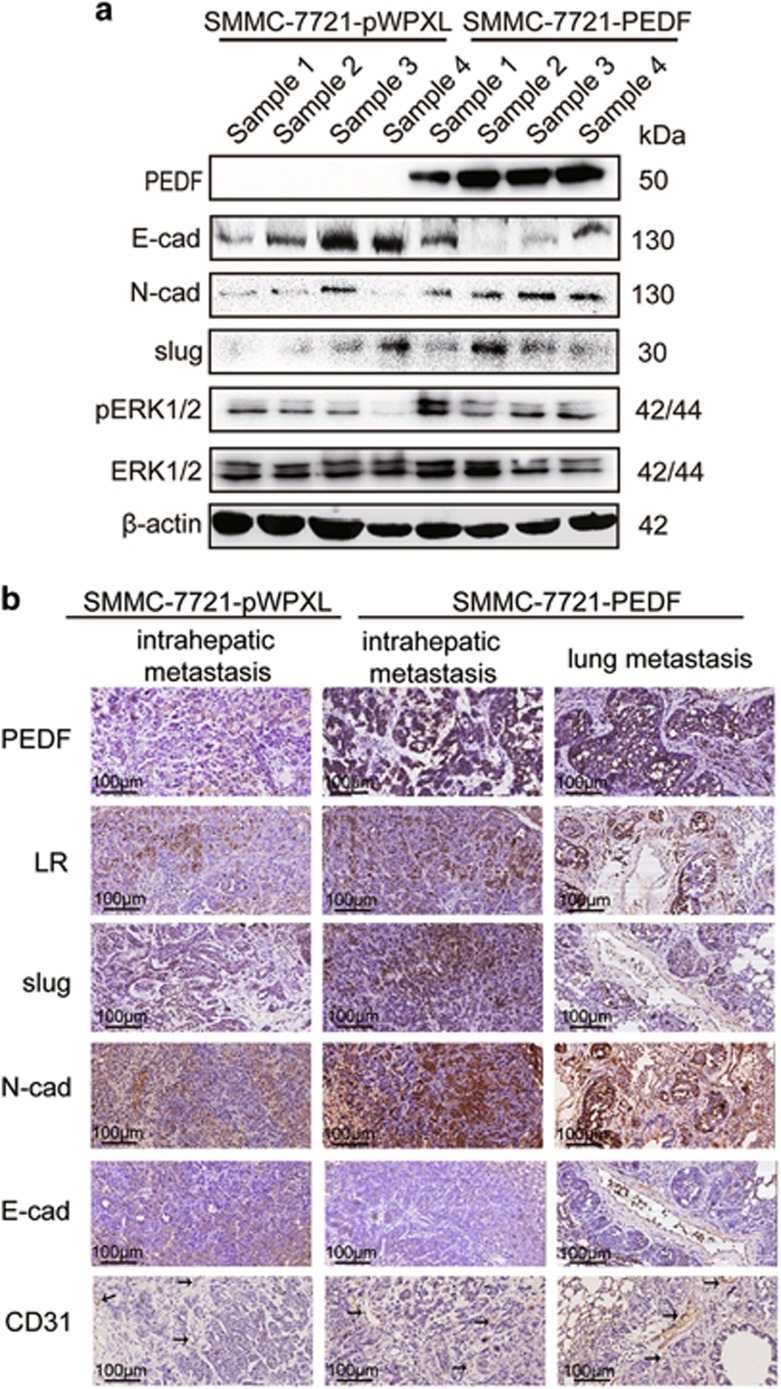
PEDF induces the change of EMT-related molecules in xenograft tissues. (**a**) The expression of E-cadherin, N-cadherin, slug, pERK1/2 and ERK1/2 was detected by western blotting in xenograft tissues. (**b**) IHC analysis of PEDF, LR, slug, N-cadherin and CD31 expression in intrahepatic metastasis tissues and lung metastasis tissues. Representative images are shown

The PI3K/AKT and ERK1/2 signaling pathways have been shown to upregulate slug, thereby causing the downregulation of E-cadherin and the upregulation of N-cadherin.^28, 29, 30^ We thus examined the possible involvement of PI3K/AKT and ERK1/2 signaling pathways in PEDF-induced EMT in HCC cells. We found that PEDF had no effect on phosphorylated AKT but upregulated the levels of Thr202/Tyr204-phosphorylated ERK1/2 (Figure 3b and Supplementary Figure 4a), suggesting that ERK1/2 signaling, as indicated by ERK1/2 phosphorylation, is involved in tumor-promoting effects induced by PEDF. To confirm the results, we used ERK1/2 inhibitors (U0126 and PD98059) to treat PEDF overexpression cells. Western blotting analysis demonstrated that U0126 (10 *μ*M) and PD98059 (30 *μ*M) significantly suppressed the levels of Thr202/Tyr204-phosphorylated ERK1/2 and reversed the EMT-related markers expression induced by PEDF (Figure 3c). We further knocked down the expression of slug in PEDF-overexpressed SMMC-7721, HCC-LY5 and PLC/PRF/5 cells. The results showed that slug depletion impaired the PEDF-induced effects on the expression of E-cadherin and N-cadherin in SMMC-7721, HCC-LY5 and PLC/PRF/5 cells (Figure 3d). Consistent with these observations, we also found that PEDF could affect the expression of EMT-related markers and the level of pERK1/2 in xenograft tissues and metastasis tissues (Figures 4a and b). IHC of CD31 in intrahepatic and lung metastasis tissues indicated that PEDF did not affect angiogenesis (Figure 4b). Therefore, these findings indicated that PEDF effectively facilitated HCC metastasis *in vitro* and *in vivo*. Thus, these data suggest that PEDF positively regulates EMT through ERK1/2 signaling pathway in HCC.

### PEDF interacts with LR in HCC cells

**Figure 5:**
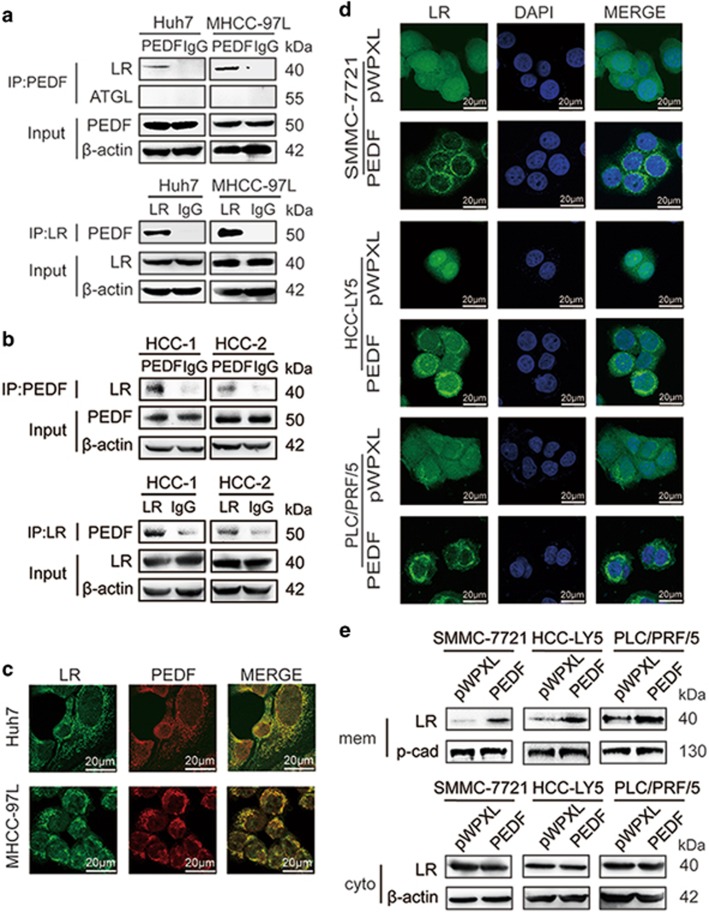
PEDF can interact with LR in HCC cells. (**a**) Co-IP analysis of PEDF interacting with LR in Huh7 and MHCC-97L cells. (**b**) Co-IP analysis of PEDF interacting with LR in HCC tissues. (**c**) Immunofluorescence staining was used to assess the colocalization of PEDF and LR in Huh7 (overlap coefficient, means±S.E.M., 0.754±0.058; *n*=3) and MHCC-97L (overlap coefficient, means±S.E.M., 0.823±0.015; *n*=3) cells. Representative images are shown. (**d**) Immunofluorescence staining was used to examine the subcellular localization of LR in PEDF-overexpressing SMMC-7721, HCC-LY5 and PLC/PRF/5 cells, and their corresponding controls. Representative images are shown. (**e**) Western blotting analysis of LR in membrane (mem) and cytosol (cyto), respectively, in PEDF-overexpressing SMMC-7721, HCC-LY5 and PLC/PRF/5 cells

Previous studies indicated that PEDF binds specifically to two receptors, namely ATGL and LR.^19^ PEDF receptors are essential for the biological effects of PEDF on various cells.^18, 19, 20^ To determine whether PEDF binds to ATGL or LR, an immunoprecipitation (co-immunoprecipitation (Co-IP)) assay was performed to identify the molecules that interact with PEDF in HCC cell lines and HCC tissues. We identified the LR, but not ATGL, in the anti-PEDF precipitated fractions, although several ATGL antibodies were used in Co-IP assays. We also detected PEDF in anti-LR precipitated samples (Figures 5a and b). Next, we conducted immunofluorescence staining to assess a potential colocalization between PEDF and LR. Our results demonstrated that PEDF and LR were strongly co-localized in Huh7 and MHCC-97L cells (Figure 5c). In addition, RT-qPCR and western blotting analysis demonstrated that PEDF overexpression did not affect the expression of LR (Supplementary Figure 5b and c), but immunofluorescence showed that PEDF overexpression increase the LR staining on the plasma membrane in PEDF-overexpressed SMMC-7721, HCC-LY5 and PLC/RPF/5 cells compared with control group (Figure 5d and Supplementary Figure 5a). We then extracted membrane protein from those cells and found that LR membrane expression was increased in PEDF overexpression cells compared with control cells (Figure 5e). Therefore, these data revealed that PEDF can interact with LR in HCC cells and affects LR by changing its subcellular localization.

### A high LR level indicates shorter survival in HCC patients and mediates the PEDF-induced migration and invasion of HCC cells

**Figure 6:**
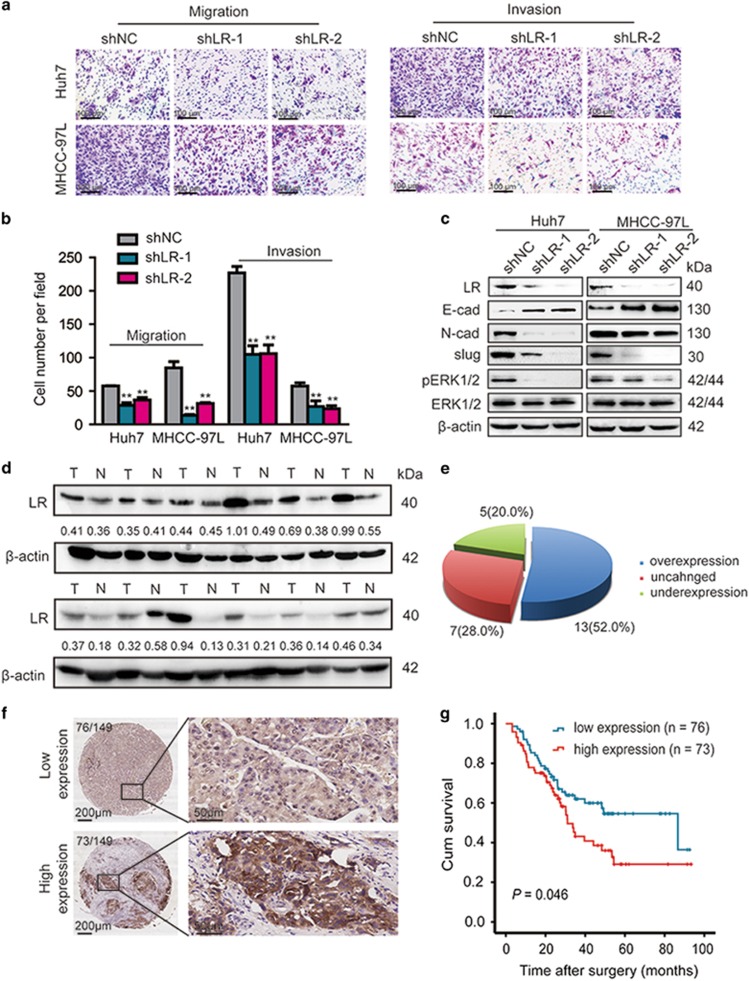
LR knockdown inhibits HCC cell migration and invasion *in vitro*, and high expression levels of LR predict a poor prognosis in HCC patients. (**a**,**b**) The *in vitro* migratory and invasive ability of LR knockdown Huh7 and MHCC-97L cells and their corresponding controls were detected and analyzed. The mean values from duplicate samples three independent experiments are shown in (**b**) (*P<0.05 and **P<0.01). (**c**) The expression of E-cadherin, N-cadherin, slug, pERK1/2 and ERK1/2 was detected by western blotting in Huh7 and MHCC-97L cells with LR shRNA. (**d**) Western blotting analysis of LR expression in HCC tissues and matched adjacent non-tumorous liver tissues. T, HCC tissues; N, adjacent non-tumorous liver tissues. (**e**) Statistical analysis of LR expression in HCC samples between HCC tissues and adjacent non-cancerous tissues. (**f**) IHC staining analysis of LR expression in 149 HCC samples. Representative images are shown. (**g**) The OS analysis of HCC patients with LR expression (*n*=149)

LR has an important role in various cancer types, where it contributes to invasion, apoptosis evasion and adhesion.^24, 31^ To test the hypothesis that LR might have a role in the PEDF-induced facilitation of HCC cell migration and invasion, we first investigated whether LR can affect the migration and invasion of HCC cells. We performed RT-qPCR and western blotting to assess LR levels in HCC cell lines and found that LR exhibited higher expression level compared with immortalized liver cell lines MIHA (Supplementary Figure 6a). Next, we knocked down endogenous LR expression in Huh7 and MHCC-97L cells using lentiviral infection (Supplementary Figure 6b). *In vitro* transwell assay results suggested that LR knockdown decreased the migratory and invasive ability of Huh7 and MHCC-97L cells (Figures 6a and b). Similarly, western blotting analysis demonstrated that LR knockdown caused the upregulation of E-cadherin and the downregulation of N-cadherin and slug (Figure 6c). The expression of LR protein expression in HCC tissues and the adjacent non-cancerous liver tissues were detected by western blotting and showed that 52.0% cases had higher LR protein expression in HCC tissues compared with the corresponding non-cancerous liver tissues (Figures 6d and e). We next analyzed the LR expression in HCC tissues using IHC. As with PEDF, all HCC patients were divided into two groups according to the expression level of LR in the tumor tissues: a high-LR-expression group and a low-LR-expression group (Figure 5f). Furthermore, the OS analysis revealed that the high expression of LR was closely associated with poor outcomes in HCC patients (*P*=0.046; Figure 6g).

**Figure 7:**
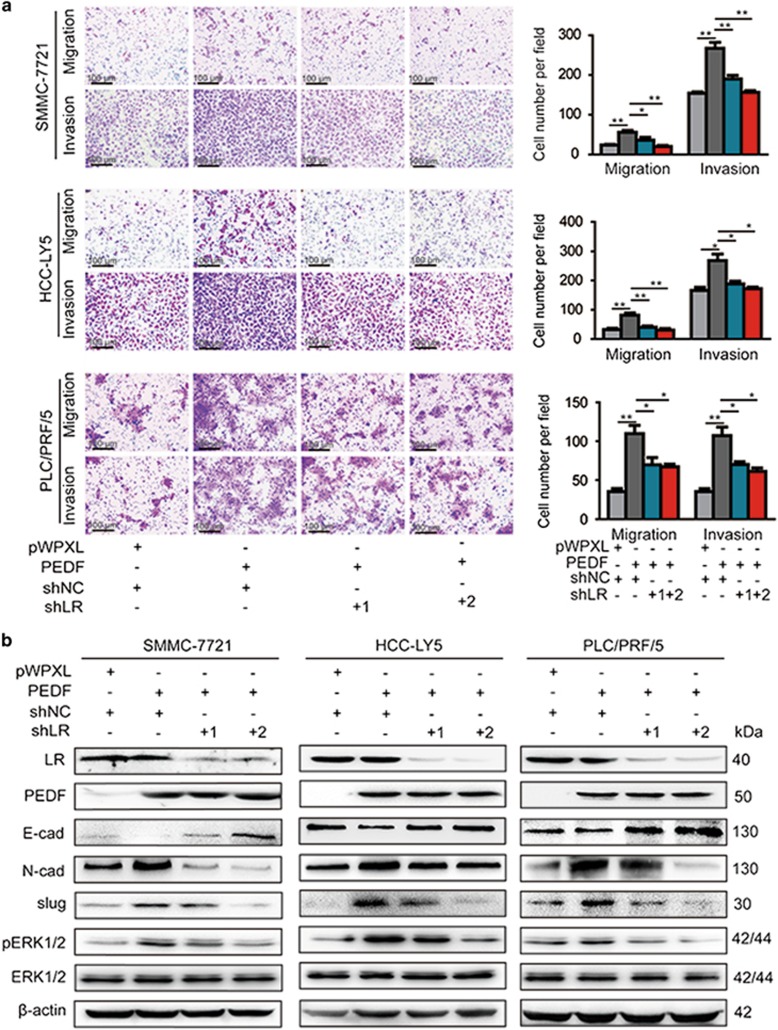
Knockdown of LR attenuates PEDF-induced HCC cell migration, invasion and EMT. (**a**) SMMC-7721, HCC-LY5 and PLC/PRF/5 cells overexpressing PEDF were transfected with shRNA targeting to LR as described. The migration and invasion ability were assessed using transwell assays. Cells transfected with the empty vector were used as a control (*P<0.05 and **P<0.01). **(b**) SMMC-7721, HCC-LY5 and PLC/PRF/5 cells overexpressing PEDF were transfected with shRNA targeting to LR as described. The expression of E-cadherin, N-cadherin, slug, pERK1/2 and ERK1/2 was detected by western blotting

To further support these findings, we knocked down the expression of LR in PEDF-overexpressed SMMC-7721, HCC-LY5 and PLC/PRF/5 cells (Supplementary Figure 6c). Consistently, PEDF-overexpressed cells were significantly more invasive and migratory in comparison to the control cells, whereas LR knockdown successfully attenuated the invasion and migration of PEDF-overexpressing SMMC-7721, HCC-LY5 and PLC/RPF/5 cells (Figure 7a). We also found that LR depletion impaired the PEDF-induced effects on the expression of E-cadherin, N-cadherin, slug and pERK1/2 in SMMC-7721, HCC-LY5 and PLC/PRF/5 cells (Figure 7b). Taken together, these results suggest that LR is an important mediator of PEDF-induced migration and invasion in HCC cells.

### PEDF expression is positively correlated with LR and N-cadherin and indicates a shorter survival time in HCC patients

**Figure 8:**
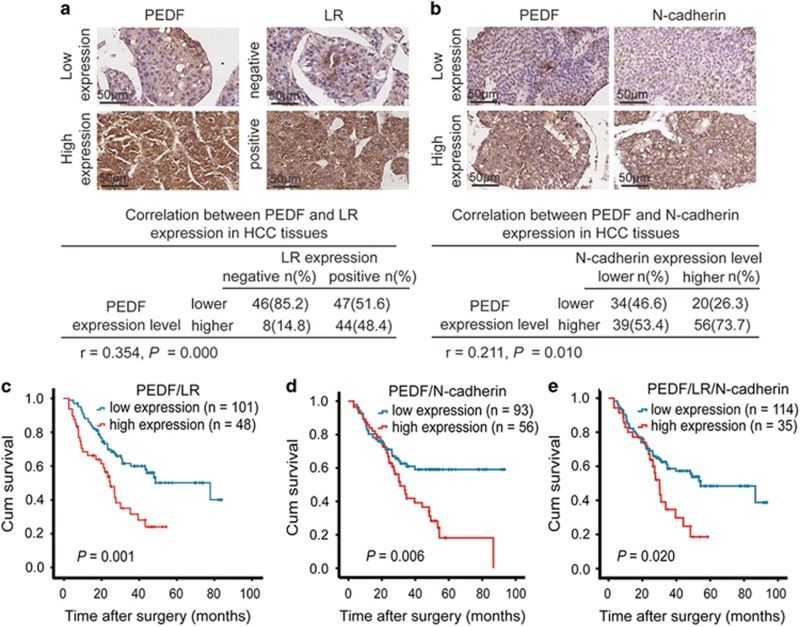
LR and N-cadherin expression is positively correlated with PEDF in HCC tissues, indicating a poor prognosis in HCC patients. (**a**) Representative images of PEDF and LR in HCC tissues are shown in the upper panel. The correlation between PEDF and subcellular localization of LR in HCC tissue is shown in the lower panel. (**b**) Representative images of PEDF and N-cadherin in HCC tissues are displayed in the upper panel. The correlation between PEDF and N-cadherin in HCC tissues is presented in the lower panel. (**c**) The OS analysis of HCC patients with high expression of PEDF and positive expression of LR. (**d**) The OS analysis of HCC patients with high co-expression of N-cadherin and PEDF. (**e**) The OS analysis of HCC patients with high co-expression of N-cadherin, PEDF and positive expression of LR. (*n*=149)

To further examine whether the interaction between PEDF and LR or the PEDF/LR-mediated regulation of EMT are clinically relevant to human HCC development, we analyzed PEDF, LR and N-cadherin expression in HCC tissues by IHC in 149 human primary samples. Based on above results, we analyzed the relationship of PEDF expression and subcellular localization of LR in HCC tissues. According to the expression and subcellular localization of LR, 149 HCC patients were divided into 2 groups: a positive expression group (Score 3, 4 and LR is predominantly expressed in membrane and cytoplasm) and a negative expression group (Score 0, 1, 2 or LR is predominantly expressed in nuclear). The analysis results showed that PEDF expression was positively correlated with positive expression of LR in HCC tissues (*r*=0.354, *P*=0.000; Figure 8a). Similarly, there was a significant correlation between PEDF and N-cadherin in HCC tissues (*r*=0.211, *P*=0.010; Figure 8b). A Kaplan–Meier survival analysis revealed that patients with high expression of PEDF and positive expression of LR had shorter OS than the low-expression group (including patients with a low expression of PEDF or negative expression of LR) (*P*=0.001; Figure 8c). Similar results were obtained for patients with high co-expression of PEDF and N-cadherin (*P*=0.006; Figure 8d). Notably, the median OS time was 34.90 (95% CI: 21.32–48.49) months for HCC patients with high expression of PEDF alone, whereas it was 27.67 (95% CI: 20.26–35.08) months for HCC patients with high expression of PEDF and positive expression of LR and 30.93 (95% CI: 23.40–38.46) months for HCC patients with high expression of both PEDF and N-cadherin. We also analyzed the prognostic value of combining PEDF, LR and N-cadherin for HCC patients and the results showed that patients with high co-expression of PEDF, N-cadherin and positive expression of LR had poorer outcomes (median OS time: 30.13 months, 95% CI: 25.62–34.64) (*P*=0.020; Figure 8e). Collectively, these data show that combining PEDF levels with subcellular localization of LR might serve to predict of poor HCC prognosis.

## Discussion

Although PEDF has an indispensable role in various types of tumors, how PEDF affects HCC progression and especially HCC metastasis has been poorly investigated. Our present study provides evidence that high expression of PEDF is associated with a poor prognosis in HCC. Although previous studies revealed the anti-tumor effects of PEDF in different tumor cells,^32, 33, 34^ recent results have shown that PEDF is overexpressed in most human hepatoma cell lines and HCC tissues, and that it is more abundant in serum samples from patients with HCC than form normal controls.^14, 17^ It has also been shown that PEDF is upregulated in melanocytic tumors.^13^ Together with our results, these findings indicate that PEDF may have opposing roles in the development and progression of cancer due to organ- or tissue-specific differences.

Our studies further demonstrated that PEDF overexpression could significantly enhance the migration and metastasis of HCC cells *in vitro* and *in vivo*. Accordingly, PEDF depletion strongly decreased the ability of HCC cells to migrate and invade. In addition, PEDF-overexpressing HCC cells exhibited a morphological change, from a rounded or cobblestone-shaped cell to a spindle-shaped, mesenchymal-like morphology and western blotting revealed that PEDF could influence the expression of E-cadherin, N-cadherin and slug. Numerous reports have indicated that EMT is a critical process in metastasis. During EMT, epithelial cells exhibit an increased capability to invade and migrate.^35^ Mounting evidence indicates that the activation of ERK1/2 positively regulates the expression of slug.^28, 29, 30^ We also observed that PEDF upregulates ERK1/2 phosphorylation. PEDF activates the ERK signaling pathway, as indicated by ERK1/2 phosphorylation in mesenchymal stem cells, primary neurons and muscle progenitor cells.^36, 37, 38^ More importantly, there was a positive correlation between PEDF expression levels and N-cadherin in HCC tissues, and a high co-expression of PEDF and N-cadherin predicted a poorer prognosis. These data suggest that PEDF induces N-cadherin and slug expression and is coupled with E-cadherin attenuation by the activation of ERK1/2, which may be involved in promoting HCC metastasis.

ATGL and LR are two putative receptors for PEDF.^12, 39^ ATGL is required for PEDF-induced lipolysis and triglyceride degradation in the liver and adipocytes.^40^ However, which receptor is mainly involved in PEDF-induced metastasis is largely unknown. In this study, we found for the first time that PEDF binds to LR but not to ATGL in HCC cells and HCC tissues. LR expression is high in numerous cancer cells, including breast, lung, gastric, prostate, colon and leukemia cells, where it indicates a worse prognosis.^41, 42, 43, 44^ In the present study, we found that high LR expression was closely associated with poor outcomes in HCC patients. In accordance with this finding, LR downregulation by shRNA significantly decreased the invasiveness of HCC cells and inhibited EMT, which suggested that LR may act as a critical tumor promoter in HCC progression.

Our results also showed that PEDF can interact with LR and LR knockdown repressed the PEDF upregulation-induced *in vitro* migration, invasion and EMT of HCC cells. It is well known that subcellular localization is important for the function of most cell surface receptors. LR is located in the plasma membrane so as to mediate cell adhesion and migration through interactions with the extracellular matrix. Lysyl-tRNA synthetase (KRS)-mediated membrane translocation of LR enhances cell migration,^45^ whereas (−)-epigallocatechin gallate (EGCG) induces a significant internalization of LR, which may serve as a mechanism underlying the inhibitory effects of EGCG on microglia activation and migration.^46^ In the present study, we found that PEDF caused change in subcellular localization of LR but did not affect the expression level of LR. Furthermore, PEDF expression was positively correlated with subcellular localization of LR in HCC tissues, and HCC patients with high PEDF expression and positive LR expression had a shorter OS than did patients with low levels of PEDF or negative LR expression. These findings support the idea that PEDF promotes migration and invasion of HCC cells via binding LR. However, it is not yet understood how PEDF induces the change in LR subcellular localization in HCC cells; this question warrants further investigation.

In conclusion, we demonstrated that PEDF has a positive role in the EMT and metastasis of HCC by interacting with LR. The combination of PEDF, LR or N-cadherin expression was more sensitive than any of them alone with respect to OS. Thus, our results suggest the PEDF-LR complex as a novel tumorigenic factor that may be a potential biomarker for the clinical prognosis of HCC and a therapeutic target in HCC.

## Materials and methods

### Reagents and cell lines

PLC/PRF/5 and HEK 293T were purchased from the American Type Culture Collection (Manassas, VA, USA); Huh7 was obtained from the Riken Cell Bank (Tsukuba, Japan); SMMC-7721 was provided by the Cell Bank of the Chinese Academy of Sciences (Shanghai, China); and the MHCC-97L cell line was obtained from Zhongshan Hospital, Fudan University (Shanghai, China). HCC-LY5 cell were established from a human primary HCC tissue in our laboratory. All cell lines were cultured in Dulbecco’s modified Eagle’s medium (DMEM) (Sigma-Aldrich, St Louis, MO) supplemented with 10% fetal bovine serum (FBS) (Hyclone, Logan, UT, USA) and were incubated at 37 °C under a humidified atmosphere with 5% CO_2_.

Antibodies used in this study are listed in Supplementary Table 2.

### Patient samples

One hundred and forty-nine human HCC tissue samples were obtained from patients who underwent surgical treatment at the Guangxi Cancer Institute (Nanning, China) and the Qidong Liver Cancer Institute (Qidong, China) between November 2001 and May 2009. The 149 HCC patients included 122 males and 27 females (mean age: 50 years, ranging from 21–83 years). No patient received preoperative chemotherapy or radiotherapy. All procedures were performed under consensus agreements and in accordance with the Chinese Ethical Review Committee.

All tissue samples were fixed in 4% phosphate-buffered neutral formalin for at least 72 h and routinely embedded in paraffin.

### Immunohistochemistry

Tissue microarrays (TMAs) that included 149 HCC tissues were constructed, and IHC staining of TMAs were performed as previously described.^47^ The results were visualized and photographed using Leica SCN400 slide scanner (Meyer Instruments, Houston, TX, USA). IHC results were scored from 0 to 4 as previously described;^48^ a score of 0–2 was considered to represent low expression and a score of 3–4 was considered to represent high expression.

### *In vitro* migration and invasion assays

Equal numbers of cells were seeded into the upper chamber of a transwell (BD Biosciences, Franklin Lakes, NJ, USA) in serum-free media. DMEM containing 10% FBS was added to the bottom chambers and the cells were incubated at 37 °C for 24 h. Cells that had migrated from the upper chamber to the lower chamber were fixed with paraformaldehyde and stained with crystal violet.

### Tumor xenograft models

Six-week-old BALB/c-nu/nu nude male mice were randomly divided into two groups. All animals were maintained under specific pathogen-free conditions; 2 × 10^6^ SMMC-7721 cells stably expressing PEDF or pWPXL-control were suspended in 40 *μ*l of a mixture of serum-free DMEM/Matrigel (1:1 volume) for each male nude mouse and orthotopically injected into the left hepatic lobe. After 4 weeks, all animals were killed. The livers and lungs were collected and fixed in 4% phosphate-buffered neutral formalin for at least 72 h and paraffin sections were prepared for hematoxylin and eosin staining.

All of the animal experiments were approved by the Institutional Animal Care and Use Committee of the Shanghai Jiao Tong University School of Medicine.

### Immunofluorescent staining

Cells were seeded onto chamber slide (Thermo Scientific, Waltham, MA, USA) for 24 h and fixed in 4% paraformaldehyde for 30 min. The dishes were then incubated with primary antibody in blocking solution overnight at 4 °C in a humidified chamber. Then dishes were washed three times in 0.5‰ PBST. After being washed, the dishes were incubated in Alexa 594-conjugated secondary antibody or 488-conjugated secondary antibody for 40 min and 4’6-diamino- 2-phenylindole in blocking solution for 20 min at 37 °C in a humidified chamber. Images were obtained with a fluorescence microscope. Colocalization relationship was analyzed by calculating Pearson’s and Spearman’s coefficients using Image-pro plus (Media Cybernetics, Rockville, MD, USA) colocalization plugin.

### Co-IP assays

Co-IP assays were performed using Huh7 and MHCC-97L cells, and HCC tissues. The cells and tissues were suspended in RIPA (Santa Cruz Biotechnology, Santa Cruz, CA, USA) lysis buffer for 40 min on ice and centrifuged at 12 000 *g* for 10 min. Antibodies against PEDF, LR and ATGL (Supplementary Table 2) were added with protein A and G agarose beads (Sigma-Aldrich), and incubated overnight at 4 °C. After washing, the complexes were subjected to western blotting analysis.

### Membrane and cytosolic protein extraction

Membrane protein extracts were collected according to the manufacturer’s instructions (Merck Millipore, Temecula, CA, USA) and protein concentration was estimated using the BCA method. The purity of membrane protein and cytosolic protein was checked by western blotting for pan-cadherin and *β*-actin antibody.

### Statistical analysis

Statistical analyses were conducted using SPSS 16.0 software. The results are presented as the mean±S.D. and two-group comparisons were evaluated using Student’s *t*-test. A survival analysis was performed using the Kaplan–Meier method. Statistical computations were performed using GraphPad version 5.0 (GraphPad Software, La Jolla, CA, USA). A bivariate correlation analysis was performed using the Pearson’s correlation method; *P* <0.05 was considered significant. *P<0.05 and **P <0.01.
