# Correction: Pigment epithelium-derived factor promotes tumor metastasis through an interaction with laminin receptor in hepatocellular carcinomas

**DOI:** 10.1038/s41419-021-03975-3

**Published:** 2021-07-20

**Authors:** Jianjing Hou, Chao Ge, Meiling Cui, Tengfei Liu, Xiaoqin Liu, Hua Tian, Fangyu Zhao, Taoyang Chen, Ying Cui, Ming Yao, Jinjun Li, Hong Li

**Affiliations:** 1 State Key Laboratory of Oncogenes and Related Genes, Shanghai Cancer Institute, Renji Hospital, Shanghai Jiaotong University School of Medicine, Shanghai, 200032 China; 2 Heze Municipal Hospital, Shandong, 274031 China; 3 Department of Etiology, Qi Dong Liver Cancer Institute, Qi Dong, 226200 Jiangsu Province China; 4 Cancer Institute of Guangxi, Nanning, 530027 China

Correction to: *Cell Death and Disease* 10.1038/cddis.2017.359, published online 3 August 2017

Following publication of this article it was noted that there were errors in figures 4 and 6. In figure 4a, the β-actin control was the same as the control used in Figure 6d, in addition, the ERK1/2 in Figure 4a was flipped horizontally. In Figure 6a, two of the migration images of MHCC-97L cells were chosen by mistake, and were the same as two of the invasion images of the Huh 7 images in Figure 6a. The corrected images are shown below. This correction does not change the description, interpretation, or the original conclusions of the manuscript. The authors apologize for any inconvenience caused.

**Figure:**
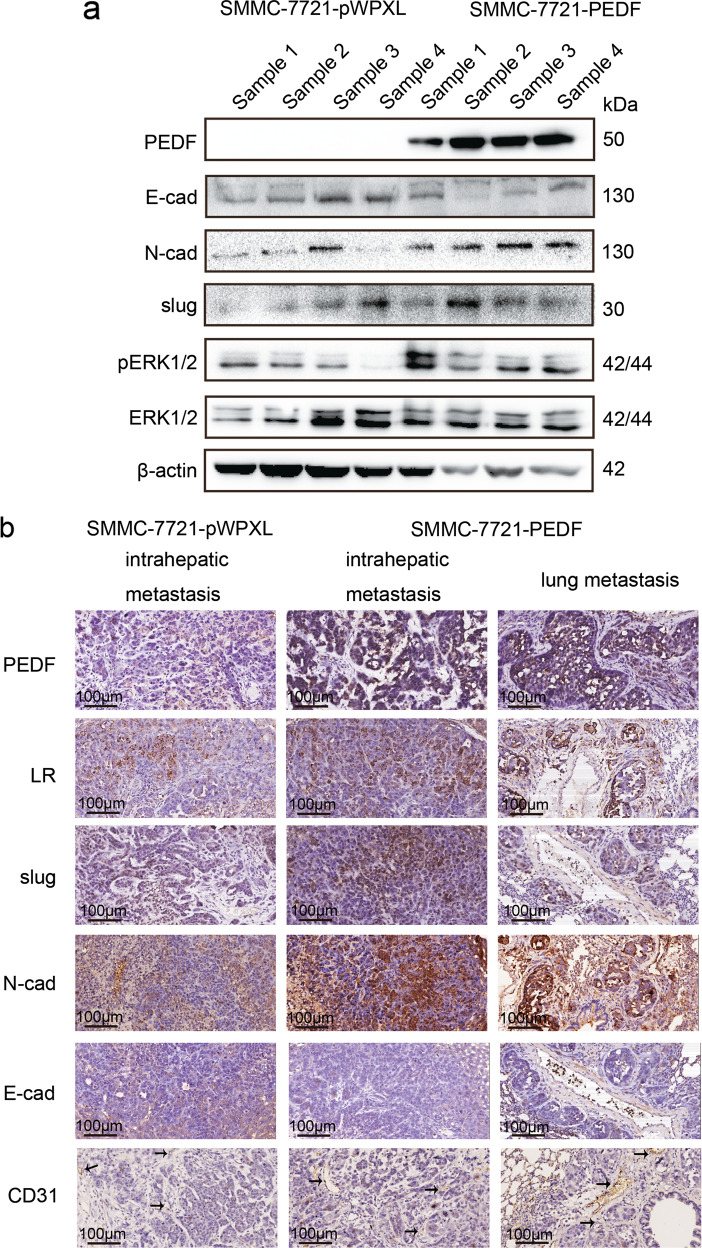
Fig. 4.

**Figure:**
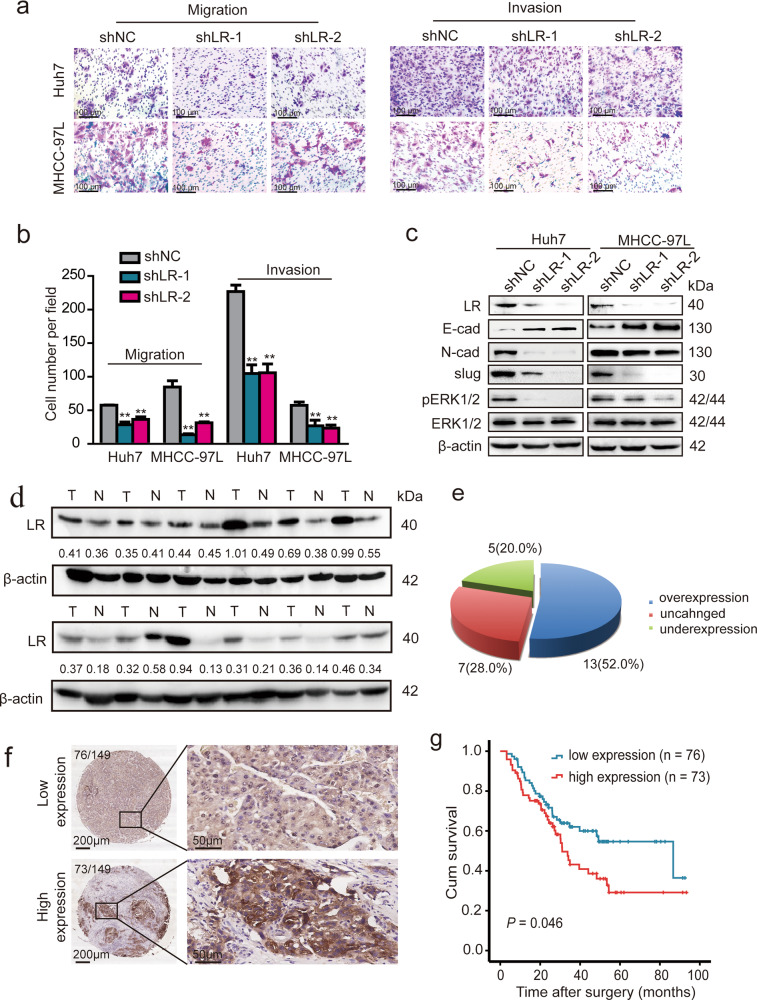
Fig. 6.

