# Supplementary material for: Pigment epithelium-derived factor promotes tumor metastasis through an interaction with laminin receptor in hepatocellular carcinomas
Source: Cell Death Dis. 2017 Aug 3;8(8):e2969–. doi: 10.1038/cddis.2017.359 (PMC5596550; doi:10.1038/cddis.2017.359)
Supplement: Supplementary Information [file cddis2017359x1.pdf]

Supplementary information

Pigment epithelium-derived factor promotes tumor metastasis through an interaction with laminin receptor in hepatocellular carcinomas

Jianjing Hou [1], Chao Ge [1], Meiling Cui [2], Tengfei Liu [1], Xiaoqin Liu [1], Hua Tian [1], Fangyu Zhao [1], Taoyang Chen [3], Ying Cui [4], Ming Yao [1], Jinjun Li [1], Hong Li [1 *]

[1] *State Key Laboratory of Oncogenes and Related Genes, Shanghai Cancer Institute, Renji Hospital, Shanghai Jiaotong University School of Medicine, Shanghai, China*

[2] *Heze Municipal Hospital, Shandong, China*

[3] *Qi Dong Liver Cancer Institute, Qi Dong, Jiangsu province, China*

[4] *Cancer Institute of Guangxi, Nanning, China*

** Corresponding author: hongli@shsci.org*

Corresponding author: Hong Li, Ph.D., State Key Laboratory of Oncogenes and Related Genes, Shanghai Cancer Institute, Renji Hospital, Shanghai Jiaotong University School of Medicine, 25/Ln 2200, Xietu Road, Shanghai 200032, China.

Tel: +86-21-64436627, Fax: +86-21-64432140, E-mail: hongli@shsci.org

Supplementary Materials and Methods

Reagents

ERK1/2 specific inhibitor U0126 and PD98059 was purchased from Sigma-Aldrich (St Louis, MO). Cells were exposed to U0126 (10 μM) or PD98059 (30 μM) or DMSO (1‰ final concentration) for 4 h.

Plasmid constructs

The PEDF sequences were PCR amplified and cloned into pWPXL (Addgene, Cambridge, MA) by replacing the GFP fragment. The primers used for cloning are listed in Supplementary Table 3.

RNA interference-based gene knockdown experiment

The short hairpin RNA (shRNA) targeting PEDF, LR and a negative control (Cat. No. GIEL2481103303) were purchased from Genechem (Shanghai, China). The small interfering RNA targeting slug and negative control were purchased from GenePharma (Shanghai, China). Different fragments were designed to target the corresponding gene transcripts, and the silencing effects of the sequences were validated by western blotting. The effective shRNA and siRNA sequences are shown in Supplementary Table 4, 5 and 6.

Western blotting

Total proteins were extracted from cell lysates and separated by 10% sodium dodecyl sulfate-polyacrylamide gel electrophoresis (SDS-PAGE). The proteins were then transferred to polyvinylidene difluoride (PVDF) membranes (Sigma-Aldrich, St. Louis, MO). The electroblotted membranes were blocked with 5 % non-fat milk and then probed with primary antibodies overnight at 4°C. After being washed, the membranes were incubated with secondary antibodies. β-actin (Sigma-Aldrich, St. Louis, MO) levels were used as an internal control.

Quantitative real-time PCR

Total RNA was isolated from cells using TRIzol reagent (Invitrogen, Carlsbad, CA, USA). Reverse transcription was performed using the PrimeScript RT Reagent Kit (Perfect Real Time) (TaKaRa Biotechnology, Shiga, Japan) according to the manufacturer’s instructions. Primers for the quantitative RT-PCR are provided in Supplementary Table 7.

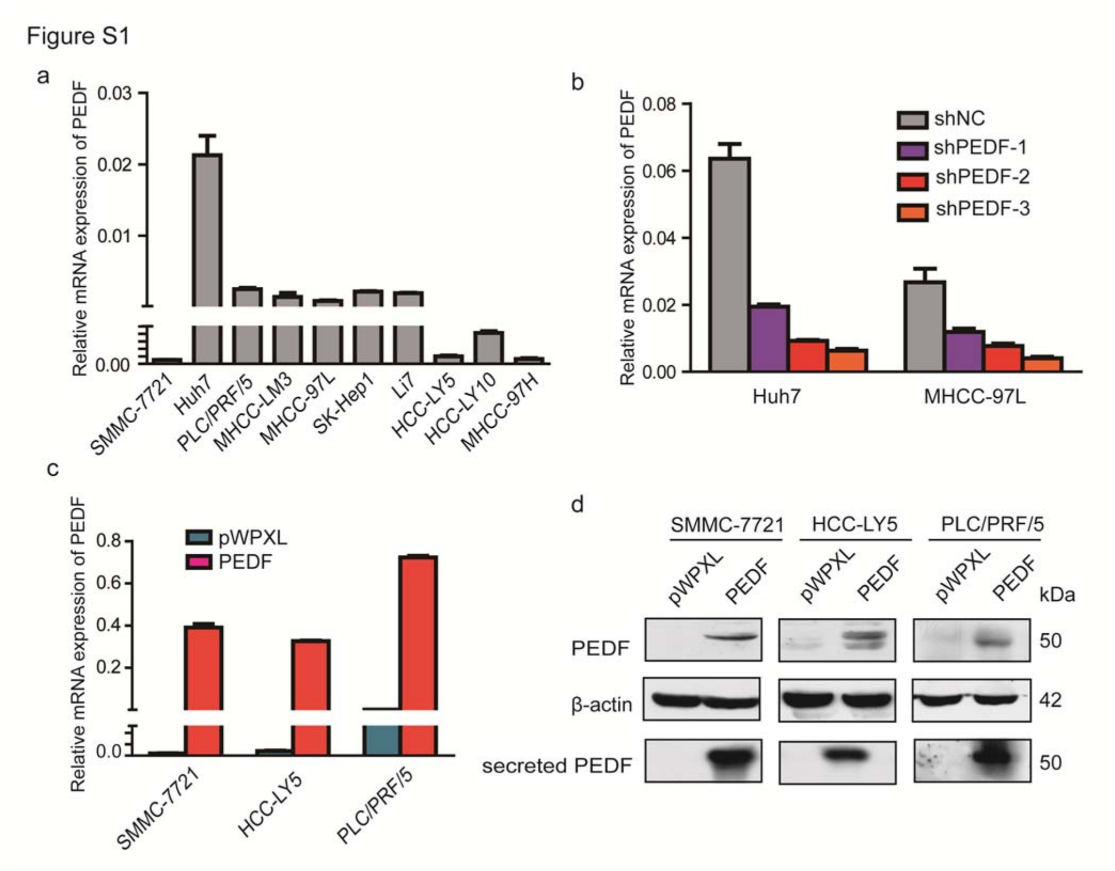

Figure S1 PEDF expression in HCC cell lines. (**a**) RT-qPCR was conducted to detect PEDF mRNA levels in HCC cell lines. (**b**) PEDF mRNA levels were detected by RT-qPCR in Huh7 and MHCC-97L cells lentivirally transduced with shPEDF and corresponding control.(**c**) The mRNA levels of PEDF were detected by RT-qPCR in SMMC-7721, HCC-LY5 and PLC/PRF/5 cells after infection with PEDF or corresponding control lentivirus. (**d**) PEDF protein was detected by western blotting analysis in SMMC-7721, HCC-LY5 and PLC/PRF/5 cells after infection with PEDF or corresponding control lentivirus.

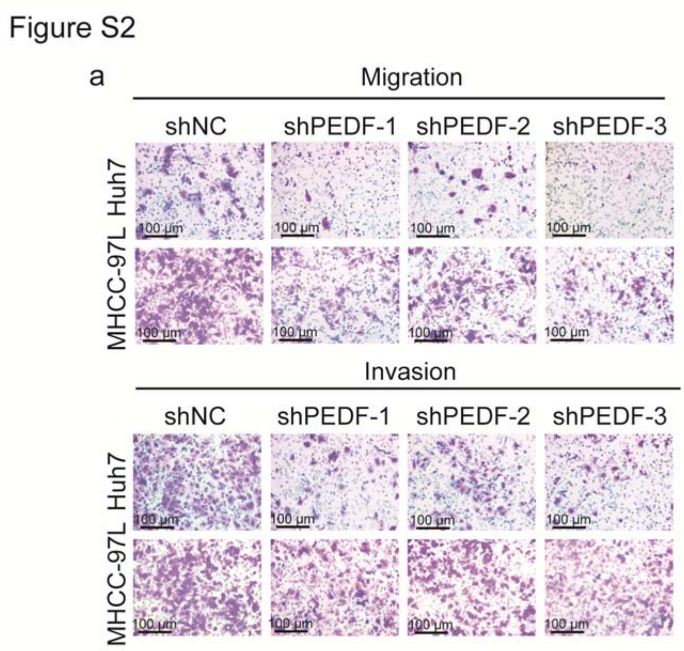

Figure S2 Knockdown of PEDF inhibited HCC cell migration and invasion *in vitro*. (**a**) The *in vitro* migration and invasion ability of Huh7 and MHCC-97L cells lentivirally transfected with shPEDF were assessed using a transwell assay; shNC was used as the control.

Figure S3

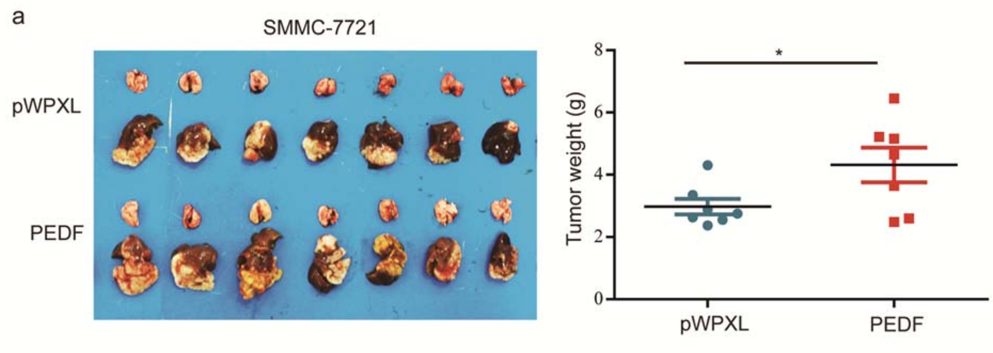

Figure S3 PEDF promoted HCC cell proliferation *in vivo*. (**a**) SMMC-7721 cells stably overexpressing PEDF were injected orthotopically into nude mice; empty vectors were used as a corresponding control. The tumors were removed from the nude mouse after 4 weeks. Representative images are shown, alone with the weight of the livers with tumors. *, $P < 0.05$.

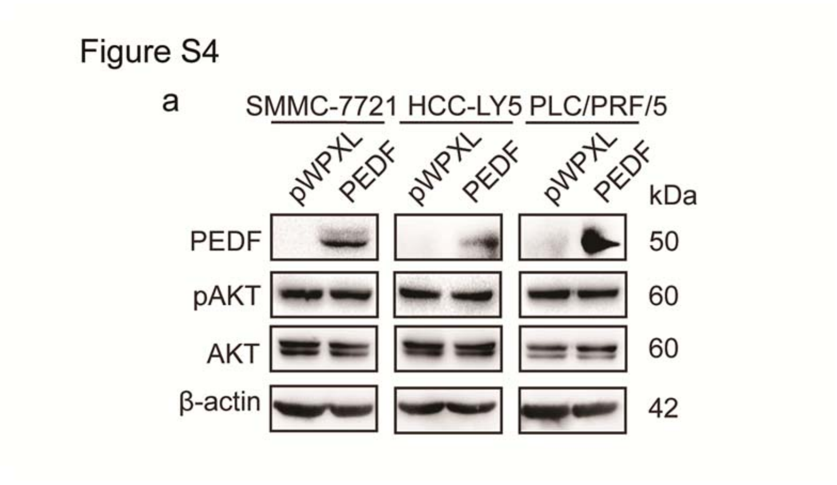

Figure S4 AKT signaling pathways in PEDF overexpression HCC cells. (a) Western blotting analysis of pAKT and AKT in PEDF-overexpressing SMMC-7721, HCC-LY5 and PLC/PRF/5.

Figure S5

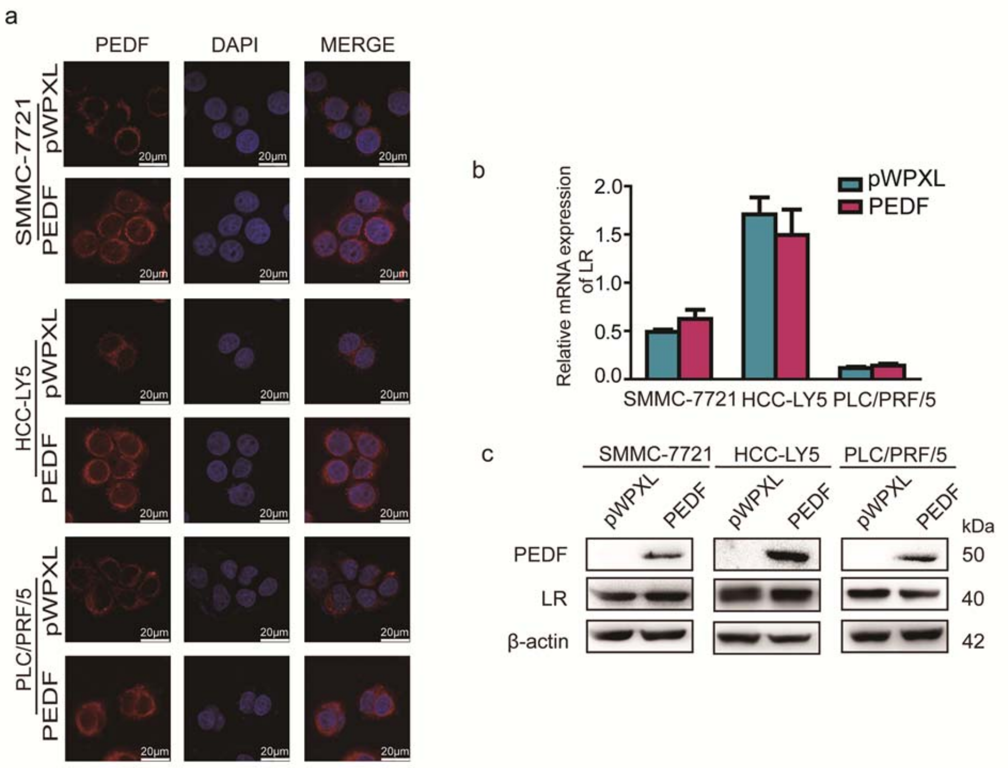

Figure. S5 (**a**) Immunofluorescence staining was used to examine PEDF expression in PEDF-overexpressing SMMC-7721, HCC-LY5 and PLC/PRF/5 cells**.** Representative images are shown. (**b**) LR mRNA levels were detected by RT-qPCR in PEDF-overexpressed SMMC-7721, HCC-LY5 and PLC/PRF/5 cells. (**c**) LR protein levels were detected by western blotting in PEDF-overexpressing SMMC-7721, HCC-LY5 and PLC/PRF/5 cells.

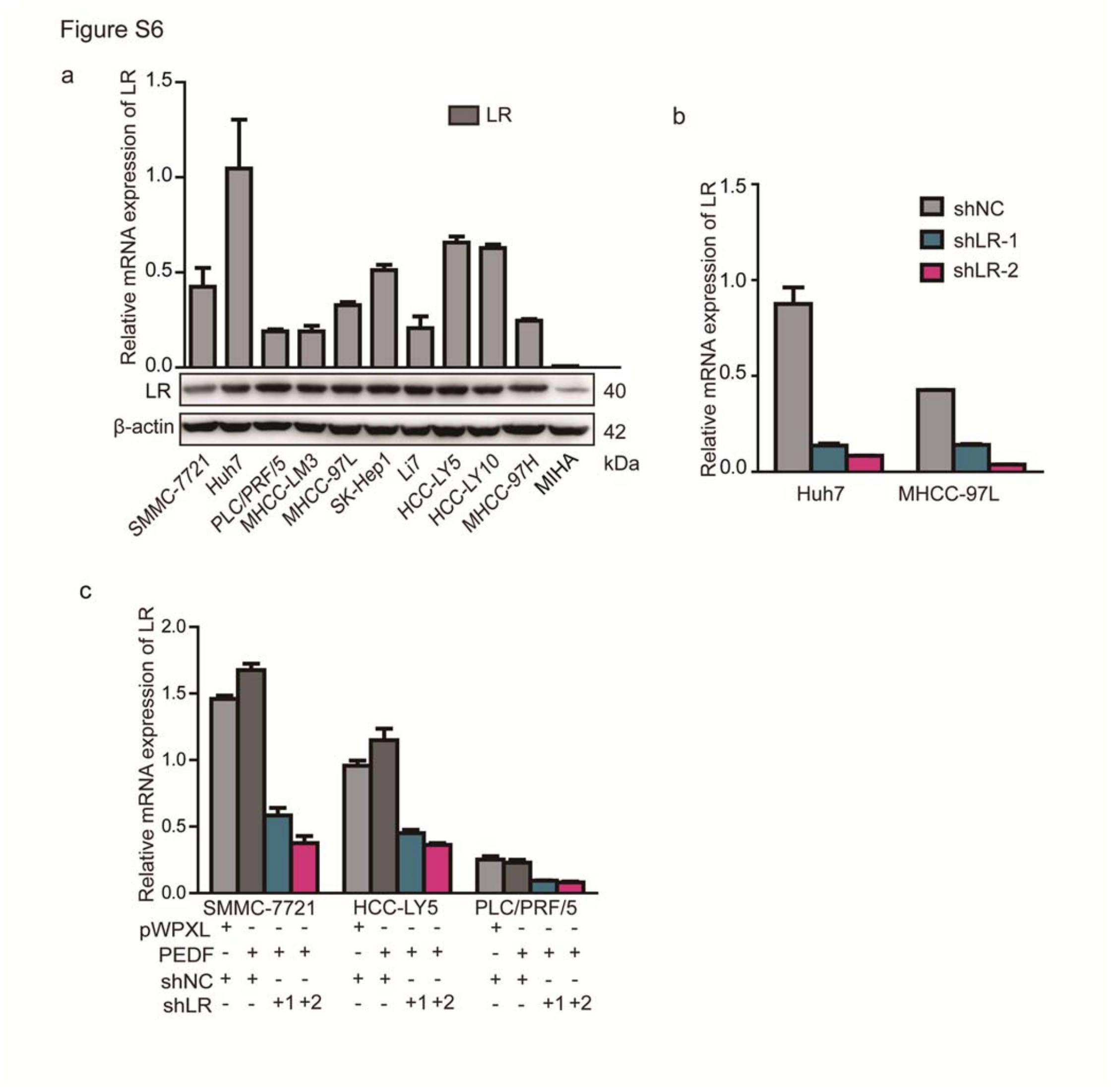

Figure S6 LR expression in HCC cell lines. (**a**) RT-qPCR and western blotting was conducted respectively to detect mRNA and protein levels of LR in HCC cell lines and immortalized liver cell line MIHA. (**b**) LR mRNA levels were detected by RT-qPCR in Huh7 and MHCC-97L cells lentivirally transduced with shLR. (**c**) PEDF-overexpressing SMMC-7721, HCC-LY5 and PLC/PRF/5 cells were lentivirally transduced with shRNA targeted to LR as indicated. The mRNA levels of LR were detected by RT-qPCR.

Supplementary Table 1 Relationship between PEDF protein expression and clinicopathological features in HCC tissues

Clinicopathological Features	Number of cases	PEDF expression Low N (%)	PEDF expression High N (%)	*P* Value
Age (years)				
≤60	121	46(85.20)	75(79.80)	0.413
>60	27	8(14.80)	19(20.20)	
Gender				
Male	122	41(75.90)	81(85.30)	0.155
Female	27	13(24.10)	14(14.70)	
Tumor size				
≤5cm	72	25(46.30)	47(52.80)	0.450
>5cm	71	29(53.70)	42(47.20)	
AFP (ng/ml)				
≤20	52	18(33.30)	34(36.60)	0.693
>20	95	36(66.70)	59(63.40)	
HBV infection				
Negative	25	9(17.60)	16(17.40)	0.969
Positive	118	42(82.40)	76(82.60)	
Cirrhosis				
Absent	12	3(5.60)	9(9.50)	0.398
Present	137	51(94.40)	86(90.50)	

Histological grade				
I, II	71	21(38.90)	50(52.60)	0.106
III, IV	78	33(61.10)	45(47.40)	
Venous invasion				
Absent	133	50(92.59)	83(87.37)	0.322
Present	16	4(7.41)	12(12.63)	
Extra-hepatic metastasis				
Absent	146	54(100.00)	92(97.87)	0.280
Present	2	0(0.00)	2(2.13)	
Lymph node metastasis				
Absent	145	54(100.00)	91(96.81)	0.185
Present	3	0(0.00)	3(3.19)	

AFP, alpha-fetoprotein. N, Number of cases. *P* value represents the probability from a Chi-square test for different immunohistochemical scores of PEDF in HCC tissues.

Supplementary Table 2 Antibodies used and dilutions

Antibody	Source	Catalogue number	Dilution	Application	Company
Primary antibodies for WB/CO-IP					
PEDF	Mouse IgG	sc390172	1:100	WB	Santa Cruz
LR	Mouse IgG	sc74531	1:100	WB	Santa Cruz
N-cadherin	Mouse IgG	Ab124397	1:500	WB	Abcam
E-cadherin	Rabbit IgG	sc7870	1:300	WB	Santa Cruz
Slug	Rabbit IgG	CST9585	1:300	WB	Cell signaling
pERK	Rabbit IgG	CST9018	1:1000	WB	Cell signaling
ERK	Rabbit IgG	CST4348	1:1000	WB	Cell signaling
β-actin	Mouse mAb	A1978	1:10000	WB	Sigma-Aldrich
PEDF	Mouse IgG	sc390172	1ug/mg protein	CO-IP	Santa Cruz
LR	Rabbit IgG	Sc20979	1ug/mg protein	CO-IP	Santa Cruz
ATGL	Rabbit IgG	MA5-14990	1:50	CO-IP	ThermoFisher SCIENTIFIC
ATGL	Goat IgG	Sc50222	1ug/mg protein	CO-IP	Santa Cruz
Secondary antibodies for WB/CO-IP					

HRP-anti-Mouse IgG	goat	A0545	1:3000	WB	Sigma-Aldrich
HRP-anti-Rabbit IgG	goat	Sc-2060	1:4000	WB	Santa Cruz
Anti-Mouse IgG HRP	Rabbit	34519	1:1000	CO-IP	ROCKLAND
Anti-Rabbit IgG HRP	Mouse	34665	1:1000	CO-IP	ROCKLAND
Anti-Goat IgG HRP	Goat	33924	1:1000	CO-IP	ROCKLAND
Primary antibodies for IHC					
PEDF	Mouse IgG	sc390172	1:10	IHC	Santa Cruz
LR	Rabbit IgG	sc20979	1:25	IHC	Santa Cruz
N-cadherin	Rabbit IgG	NB600-1038	1:100	IHC	Novus
E-cadherin	Rabbit IgG	sc7870	1:10	IHC	Santa Cruz
slug	Rabbit IgG	CST9585	1:100	IHC	Cell signaling
CD31	Rabbit IgG	ab28364	1:25	IHC	Abcam
Secondary antibodies for IHC					
EnViSion (Peroxidase/DAB; Rabbit/mouse)	Detection	Kit K5007		IHC	DAKO

Supplementary Table 3 Primers for Cloning plasmid

Name	Primer Sequence (5’-3’)
PEDF-F	CGACGCGTATGCAGGCCCTGGTGCTACTCC
PEDF-R	GGAATTCTTAGGGGCCCCTGGGGTCCA

Supplementary Table 4 Sequences used for to silence PEDF

Name	Primer Sequence (5’-3’)
shNC	TTCTCCGAACGTGTCACGT
shPEDF-1	TGTTTGATTCACCAGACTT
ShPEDF-2	GAAGCATGAGTATCATCTT
shPEDF-3	AGCGAACAGAATCCATCAT

Supplementary Table 5 Sequences used for to silence LR

Name	Primer Sequence (5’-3’)
shNC	TTCTCCGAACGTGTCACGT
shLR-1	TCTGTACTTCTACAGAGAT
shLR-2	TGCAATTGTTGCCATTGAA

Supplementary Table 6 Sequences used for to silence slug

Name	Primer Sequence (5'-3')
siNC	UUCUCCGAACGUGUCACGUTT
sislug-1	GCUUCAAGGACACAUUAGATT
sislug-2	CCUGCACAAACAUGAGGAATT

Supplementary Table 7 Primer for the Real-time PCR

Name	Primer Sequence (5'-3')
PEDF-F	CCTTGACCGGAAGCATGAGT
PEDF-R	GGATTGCAGCTTCATCTCCTG
LR-F	TGCAGCAGGAACCCACTTAG
LR-R	GCAAACTTCAGCACAGCCCT
